# Accelerated tibial fracture union in the third trimester of pregnancy: a case report

**DOI:** 10.1186/1752-1947-2-44

**Published:** 2008-02-09

**Authors:** Mudussar A Ahmad, Damayanthi Kuhanendran, Irvine W Kamande, Charalambos Charalambides

**Affiliations:** 1 Department of Trauma & Orthopaedics, The Whittington University Hospital, London, UK

## Abstract

**Introduction:**

We present a case of accelerated tibial fracture union in the third trimester of pregnancy. This is of particular relevance to orthopaedic surgeons, who must be made aware of the potentially accelerated healing response in pregnancy and the requirement for prompt treatment.

**Case presentation:**

A 40 year old woman at 34 weeks gestational age sustained a displaced fracture of the tibial shaft. This was initially treated conservatively in plaster with view to intra-medullary nailing postpartum. Following an emergency caesarean section, the patient was able to fully weight bear without pain 4 weeks post injury, indicating clinical union. Radiographs demonstrated radiological union with good alignment and abundant callus formation. Fracture union occurred within 4 weeks, less than half the time expected for a conservatively treated tibial shaft fracture.

**Conclusion:**

Long bone fractures in pregnancy require clear and precise management plans as fracture healing is potentially accelerated. Non-operative treatment is advisable provided satisfactory alignment of the fracture is achieved.

## Introduction

Tibial fractures are the second most common long bone fracture. Treatment varies according to fracture displacement, complexity and whether the fracture is open or closed. The options are non-operative treatment, with plaster immobilization and traction, or operative treatment, with intra-medullary nailing, plating and external fixation. The potential complications of non-operative treatment include delayed union, mal-union and non-union. Operative management has similar complications with the addition of wound infection, osteomyelitis and fat embolism.

Surgical intervention in pregnancy presents a risk to the foetus. However surgery can be successfully performed when a multidisciplinary team approach is used [1].

Fracture healing occurs in three phases: inflammatory, reparative and remodelling [2]. This is a dynamic process which is mainly regulated by local interactions among cells and tissues around the fracture site. Tissue repair is also influenced by hormones that act systemically, such as insulin and glucocorticoid, and gonadal hormones, such as oestrogen and androgens [3], which are all increased in pregnancy.

Accelerated union of fractures has been seen in children and in patients with head injuries, neurological disease (e.g. spina bifida, paraplegia) and burns.

We present a case of accelerated tibial fracture union in a pregnant woman.

## Case presentation

**Figure 1:**
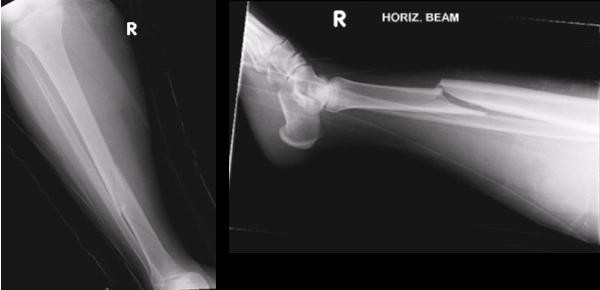
Initial radiographs.

A 40 year old obese African woman (weight 135 kg) who was 34 weeks pregnant injured her right leg following a fall in the bathroom. Previous medical history included thalassaemia trait and severe bipolar affective disorder which was being treated with Lithium Carbonate and prochlorperazine. She was a non-smoker and did not drink alcohol. On examination the leg was swollen, slightly deformed with the skin intact and there was no neurovascular deficit or evidence of compartment syndrome. Radiographs of the tibia revealed a displaced oblique mid-shaft fracture of the right tibia, 42-A2.1 using the AO classification (fig. 1).

The initial plan was non-operative treatment until postpartum, after which the fracture would be stabilised by an intra-medullary nail. She was admitted to hospital and a below knee backslab followed by a full Sarmiento cast applied. An above knee plaster could not be applied due to thigh bulk. The patient was allowed to touch weight bear for nursing purposes. Our main concern regarding the non-operative management in a plaster cast was the increased risk of developing a deep vein thrombosis. At 38 weeks of pregnancy, an emergency caesarean section was performed and a healthy baby delivered.

**Figure 2:**
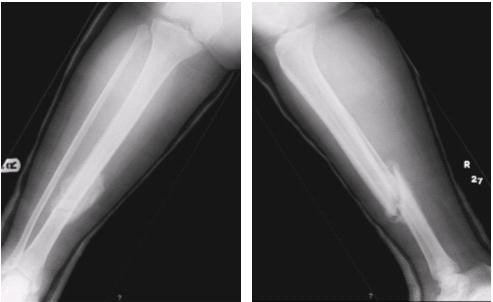
Radiograph following caesarean section, 4 weeks post injury.

**Figure 3:**
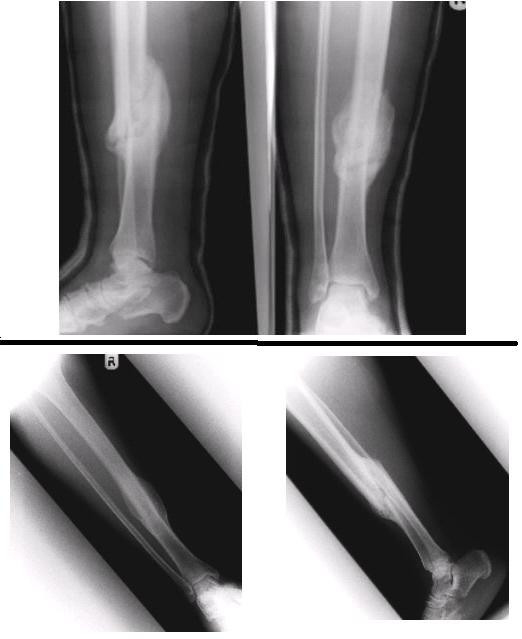
Radiograph 8 weeks and 2 years post injury.

Prior to the planned surgery in the post-natal period, it was noticed that the patient was able to mobilise with full weight bearing through the plaster without pain. Clinical examination revealed no pain or movement at the fracture site indicating clinical union. Radiographs at four weeks (fig. 2) showed satisfactory alignment and significant callus bridging all four cortices indicating radiological union. The patient was allowed to fully mobilise as tolerated in an air cast boot and reviewed in four weeks with a further radiograph that showed a consolidated fully healed fracture (fig. 3).

Review two years post injury showed a united fracture (fig. 3). The patient was asymptomatic with no clinical deformity and a full range of pain free motion in her ankle and knee.

## Discussion

Fracture healing is influenced by factors related to the injury and those related to the patient. Factors related to the injury include whether the fracture is open or closed, the severity of soft tissue injury, the degree of contamination in cases of open fracture and the adequacy of reduction. Patient factors include age, smoking, alcohol intake and the use of medications such as steroids or non-steroidal anti-inflammatory drugs.

In this case, we propose that the main contributing factor for accelerated union by four weeks is most likely hormonal. In pregnancy, there is an increase in the level of steroid hormones, initially with progesterone in the first trimester followed by the oestrogens and prolactin in the 2^nd ^and 3^rd ^trimesters [4]. Oestrogen has well-documented effects on bone formation and remodelling during fracture healing [5]. Radioligand binding studies in a fibula osteotomy (created fracture) model of fracture healing in New Zealand rabbits demonstrated the presence of oestrogen receptors in fracture sites in a bimodal distribution with a peak occurring on day 16 post-osteotomy [6]. Oestrogen receptors have been shown to be present in fracture callus [7]. It has also been shown that treating ovariectomized rats with oestrogen during fracture healing strengthens the healing callus and increases expression of cartilage matrix proteins [8]. This suggests high levels of oestrogen at this specific time post fracture would have a maximal effect on bone healing as the oestrogen receptors in callus are also maximal at this stage. The hyperdynamic circulation in pregnancy may also contribute to accelerated fracture healing by delivering the cellular factors and hormones to the fracture site at a faster rate. A significant increase in heart rate can be demonstrated as early as the 5th week in pregnancy and this contributes to an increase in cardiac output at this time [9]. There is a progressive augmentation of stroke volume (10–20 ml) during the first half of pregnancy, probably related to incremental changes in plasma volume and as a consequence cardiac output increases from an average of under 5 l/min before pregnancy to approximately 7 l/min at the 20th week of pregnancy [9]. This results in a faster delivery of cellular factors and hormones to the fracture site.

This woman probably mobilised with full weight bearing as comfort allowed in the plaster cast, as touch weight bearing would have been unrealistic for someone weighing 135 kg. Early weight bearing has been shown to promote fracture healing and this may also have contributed to accelerated fracture union. Kenwright et al compared two groups of rigidly fixed tibial shaft fractures, one with no movement and one with axial micromovement at the fracture site (induced by weight bearing). Time to clinical union and full weight bearing was significantly less and fracture stiffness was greater in the micromovement group [10].

Tibial fractures are a complex group of injuries with many potential complications. A meta-analysis of published studies between 1966 and 1993 of three methods of treatment determining the clinical outcomes of the treatment of closed tibial shaft fractures with immobilization in a cast, open reduction with internal fixation or fixation with an intra-medullary nail revealed open reduction and internal fixation to be associated with a higher rate of bony union by twenty weeks than treatment with a cast [11].

In a prospective review of 13 studies which looked at 895 tibial shaft fractures treated by application of a plaster cast, fixation with plate and screws, and reamed or unreamed intra-medullary nailing, the combined incidence of delayed and non-union was higher with closed treatment (17.2%) in comparison to operative treatment (2.6% with plate fixation, 8.0% with reamed nailing and 16.7% with unreamed nailing) [12]. These studies suggest tibial fractures treated conservatively take longer to unite, and should usually do so by approximately 20 weeks, 12 weeks longer than in our patient.

## Conclusion

1. Long bone fractures in pregnancy require clear and precise management plans as fracture healing is potentially accelerated.

2. Non-operative treatment is advisable provided satisfactory alignment of the fracture in plaster is achieved early on.

3. If operative treatment is delayed, technical difficulties may be encountered during definitive surgery, due to the potentially accelerated healing response.

4. A better understanding of the biology of bone healing is required especially in pregnancy.

## Competing interests

The author(s) declare that they have no competing interests.

## Authors' contributions

MAA analysed the literature, results, radiographs, wrote & corrected the manuscript. DK did the literature search and compiled results. IWK compiled the radiographs and thought of the idea. CC corrected the draft of the manuscript and approved for publication. All authors read and approved the final manuscript.

## Consent

Written informed consent was obtained from the patient for publication of this case report and all accompanying images. A copy of the written consent is available for review by the Editor-in-Chief of this journal.
